# Photosynthetic Properties of *Miscanthus condensatus* at Volcanically Devastated Sites on Miyake-jima Island

**DOI:** 10.3390/plants9091212

**Published:** 2020-09-16

**Authors:** Xiulong Zhang, Hao Li, Xiaoxing Hu, Pengyao Zheng, Mitsuru Hirota, Takashi Kamijo

**Affiliations:** 1 Graduate School of Life and Environmental Sciences, University of Tsukuba, Tsukuba 305-8572, Japan; zhangxiulong471391364@gmail.com (X.Z.); leehboy@hotmail.com (H.L.); huxiaoxingcn@yahoo.co.jp (X.H.); s1921192@s.tsukuba.ac.jp (P.Z.); 2 Faculty of Life and Environmental Sciences, University of Tsukuba, 1-1-1 Tennoudai, Tsukuba, Ibaraki 305-8572, Japan; hirota0313@gmail.com

**Keywords:** volcanically devastated sites, low N, *Miscanthus condensatus*, leaf traits, photosynthetic N use efficiency

## Abstract

How photosynthetic-related leaf traits of non-nitrogen (N)-fixing pioneer species respond to extreme habitat conditions of primary succession is still not well-elucidated, especially in volcanically N-deplete habitats. The effect of N-deplete soil on photosynthetic-related leaf traits can provide a basis for predicting how plants adjust their strategies to adapt to such habitats. To examine the responses of leaf traits to extreme conditions, we investigated *Miscanthus condensatus* (a non-N-fixing C_4_ pioneer grass) which grows on a volcanically devastated area on Miyake-jima Island, Japan, in which the volcanic ash has been deposited for 17–18 years since the 2000-year eruption. Leaf N content (N_area_), light-saturated photosynthetic rate (A_max_), and photosynthetic N use efficiency (PNUE) in three contrasting study sites: bare land (BL), shrub land gap (SLG), and shrub land under canopy (SLUC) were determined. Results indicated that compared to previous studies and internal comparison of Miyake Island, *M. condensatus* in BL was able to maintain a relatively high A_max_, N_area_ and PNUE. The higher A_max_ was in part a result of the higher PNUE. This is a characteristic necessary for its successful growth in N-deplete soils. These results suggest that *M. condensatus* has photosynthetic-related advantages for adaptation to volcanically N-deplete habitats.

## 1. Introduction

Volcanic ecosystems are usually characterized by high levels of stress and disturbance [1]. In early volcanic successional systems, plant growth is severely limited by nitrogen (N) [2,3]. Plants inhabiting volcanic ecosystems have to withstand the lack of N, which limits their growth and ability to invade and establish new sites. Furthermore, surviving in volcanic devastated sites requires coordinated physiological responses [4,5,6]. Pioneer species seem to be highly adaptable to adverse environments. Many dominant pioneer species can adapt to environmental changes by adjusting their growth strategies [7,8]. For example, altered root morphology, improved resource use efficiency, resorption efficiency (e.g., N, P), and N-fixing ability are all important adaptation strategies that allow plants to cope with poor nutrient habitats. Many studies have focused on N-fixing pioneer species at new volcanically devastated sites [9,10,11,12], because N is scarce in new volcanic materials, such as lava, scoria, and volcanic ash [2]. However, there are also many pioneer species without the N-fixing ability in volcanic succession. This raises the question of how non-N-fixing pioneer species adjust their strategies to adapt to nutrient-deplete habitats. However, little research has been carried out on the physiological ecology of non-N-fixing pioneer species inhabiting harsh environments such as volcanic sites [13,14,15].

N plays a vital role in plant functioning and is one of the most important limiting nutrients in terrestrial ecosystems [2,16]. Leaf traits provide insights into the N use strategy of plants. Leaf N content (N_area_), leaf mass per area (LMA), and light-saturated photosynthetic rate (A_max_) are the main leaf traits correlated with each other [17,18]. Numerous studies have found that N_area_ is associated with abiotic factors, such as light availability [19,20], nutrient availability [21,22], temperature [23,24], fire regime [25], and volcanic gas [12]. The relationship between N_area_ and these abiotic factors reflects the adaptive response of the plant to the local environmental conditions. Many studies have demonstrated that the A_max_ and N_area_ were lower in N-deficient soil than in soil not limited in N [26,27,28]. Efficient use of N can determine the competitive ability [29] and dominance of a plant in a given environment [30]. Thus, efficient use of N is essential for pioneer species without N-fixing ability to successfully establish and grow in N-deficient habitats. Photosynthetic N-use efficiency (PNUE: the rate of net photosynthesis accomplished per unit N in a leaf), are frequently used as important leaf traits for characterizing leaf photosynthetic economics [31]. However, the pattern of change in PNUE under N-limited conditions has been inconsistent; studies have shown that PNUE values have increased [28,32], decreased [33,34] and showed no marked variation [35] along N addition gradients. In addition, PNUE is negatively correlated with LMA and increases with higher A_max_ and N_area_ levels [36,37]. However, relatively little is known about the relationships of these traits of non-N-fixing pioneer species at volcanically devastated sites.

*Miscanthus*, a genus of the Poaceae and a C_4_ plant, have a wide distribution and are found across both the cool and warm temperate regions of East Asia [38]. In Japan, two closely related species, *Miscanthus sinensis* and *M. condensatu* (neither have the N-fixing ability), are common dominant pioneer species in the early stages of volcanic succession [39,40,41,42]. In Miyake-jima Island, Japan, the site of focus in this study, *M. condensatus* was the most dominant species in the ecological recovery process following the volcanic eruption of large amounts of ash and gas in the year 2000 [42]. According to a chronosequence study on lava flow of different ages, the process of primary succession on Miyake-jima Island can be summarized as follows: (1) successful colonization of pioneer species, such as deciduous *Alnus sieboldiana* (a N-fixing tree), *M. condensatus* (a perennial grass), and *Fallopia japonica* var. hachidyoensis (a perennial herb), on bare lava flows; (2) colonization of several deciduous species such as *Prunus speciosa* and climax evergreen species such as *Machilus thunbergii* and *Castanopsis sieboldii*, and the disappearance of pioneer species; and (3) establishment of the climax forest, composed of evergreen *C. sieboldii* [9]. The successional process following the eruptions in 2000 differs from that on lava flow, and *M. condensatus*, the species of interest in this study, extensively dominated the new bare land [42]. However, to the best of our knowledge, physiological studies of other pioneer species on volcanically devastated sites are limited [3,43,44], and no research has been conducted to understand the growth strategies of these two *Miscanthus* species through physiological traits. In addition, there are no quantitative studies of the PNUE of pioneer species on volcanically devastated sites. Therefore, we hypothesized that the photosynthetic-related leaf traits, especially the PNUE in *M. condensatus,* play a role in the survival of this species, particularly when it grows in volcanically N-deplete soil.

To test this hypothesis, we examined (1) whether the *M. condensatus* displays any difference in photosynthetic-related leaf traits among different habitat conditions, and (2) how environmental factors influenced the photosynthetic-related leaf traits of *M. condensatus* in extreme volcanically devastated sites.

## 2. Results

### 2.1. Study Site Description

Among three sites, in bare land (BL) and shrub land gap (SLG), *M. condensatu* were the absolutely dominant species. The other species such as *A. sieboldiana* (N-fixing species) and *F. japonica* var. hachidyoensis (a perennial herb) were also existed sparsely. In shrub land under canopy (SLUC), *M. condensatus* was sparsely distributed under the closed canopies, and some dead *M. condensatus* were found. Soil N (STN) (0–5 cm) and canopy openness varied significantly across the three study sites. In BL, STN was significantly lower than in SLG and SLUC. (Table 1, *p* < 0.05). However, there was no significant difference in STN between SLG and SLUC (Table 1, *p* > 0.05). Canopy openness value was relatively similar between BL and SLG (higher than 60%), while canopy openness in BL was significantly higher than that in SLUC (Table 1, *p* < 0.05). The maximum daily photosynthetic photon flux density (PPFD) among the three study sites reached 1227 µmol·m^−2^·s^−1^ (BL), 531 µmol·m^−2^·s^−1^ (SLG) and 102 µmol·m^−2^·s^−1^ (SLUC) at noon, respectively (Table 1).

### 2.2. Monthly and Site Changes in Leaf Traits of M. condensatus

The main effect of site was statistically significant for most of the leaf traits of *M. condensatus* (Table 2, *p* < 0.05)*,* whereas water use efficiency (WUE) and PNUE were not affected by site. In BL, *M. condensatus* had significantly lower N_area_, A_max_, maximum quantum yield of PS II (Fv/Fm), and transpiration rate (E), but significantly higher LMA and WUE than those in the SLG or SLUC (Table 3, *p* < 0.05). Light compensation point (LCP) value in the SLUC was significantly lower than that in the BL and SLG (Table 3, *p* < 0.05), but the difference in mean between BL and SLG was not significant. In terms of PNUE, there was no significant difference in values from the three sites (Table 3, *p* > 0.05).

**Figure 1:**
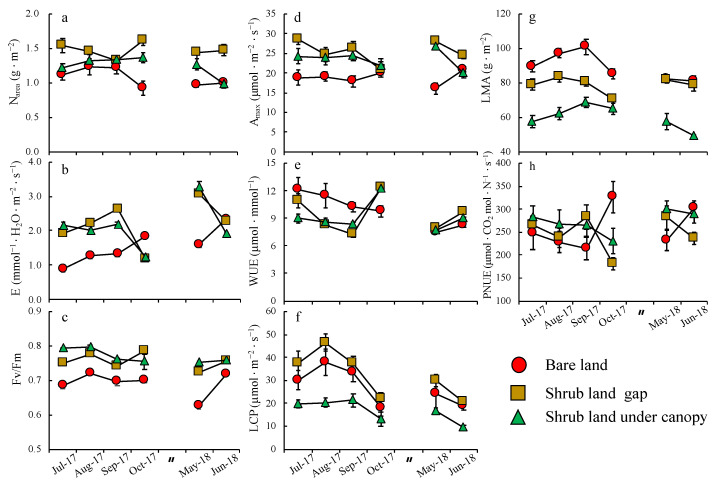
Monthly changes of leaf N content (N_area_) (**a**); transpiration rate (E) (**b**); maximum quantum yield of PSII (Fv/Fm) (**c**); light-saturated photosynthetic rate(A_max_) (**d**); water use efficiency (WUE) (**e**); light compensation point (LCP) (**f**); leaf mass per area (LMA) (**g**); photosynthetic N use efficiency (PNUE) (**h**) in bare land (BL) (circle), in shrub land gap (SLG) (square) and in shrub land under canopy (SLUC) (triangle). Vertical bars represent the mean ± SE (n = 8).

**Figure 2:**
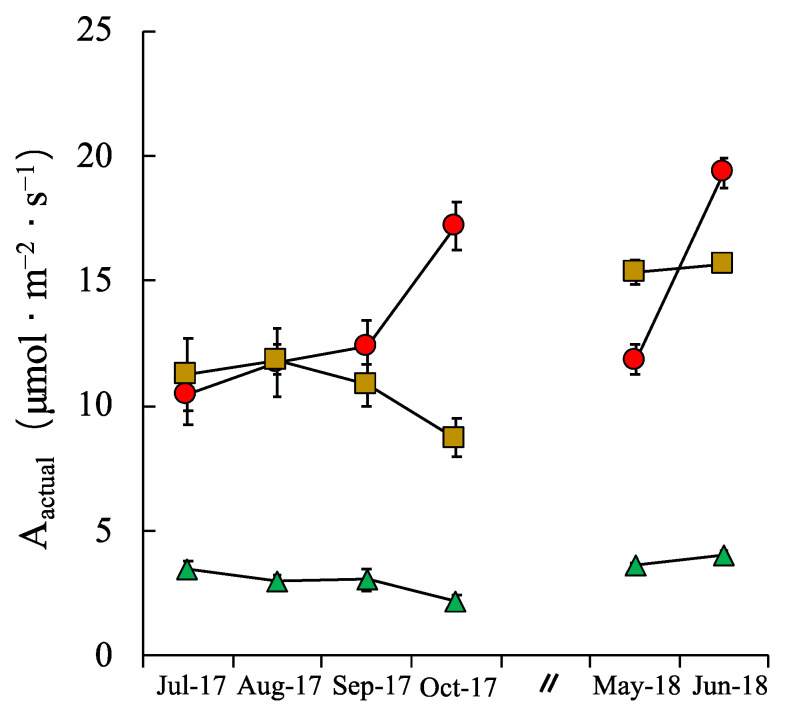
Monthly changes of estimate actual photosynthetic rates (A_actural_) under actual ambient maximum photosynthetic photon flux density (max PPFD: µmol·m^−2^·s^−1^) of *M. condensatu*s grown in BL (circle), in SLG (square) and in SLUC (triangle). Vertical bars represent the mean ± SE (n = 8).

During the growing season, Fv/Fm, E, N_area_, LMA and WUE, showed significant monthly changes (Table 2, *p* < 0.05), and relative values among the three sites varied in each month (Figure 1). The site by month interaction was significant for all leaf traits of *M. condensatus* (Table 2, *p* < 0.05). In the BL, the A_max_, E, and PNUE values in October 2017 and June 2018 were higher than those recorded in the other months (Figure 1b,d,h); also, the PNUE values in these two months were higher in the three sites (Figure 1h). Figure 2 shows the estimated actual photosynthetic rates (A_actual_) in the three sites. The A_actual_ in SLUC only reached 23–30% of the A_actual_ recorded in SLG.

### 2.3. Effect of Environment Factors on the Leaf Traits of M. condensatus

Based on the generalized linear mixed model (GLMM) results (Table 4), canopy openness had significant positive effects on the Fv/Fm, LMA and LCP. No association between canopy openness and the remaining leaf traits was found. STN had a significant positive effect on N_area_ and A_max_. The precipitation had significant positive effect on A_max_ and PNUE. As for PNUE, both canopy openness and STN significantly affected it negatively (Table 4).

## 3. Discussion

### 3.1. Advantages of M. condensatus in Leaf Traits at Miyake Volcanically Devastated Site

Our aim in this study was firstly to examine photosynthetic-related advantages of *M. condensatus* in volcanically devastated sites. Normally, N_area_ for C_4_ plants is considered to range from 1.68 to 2.52 g·m^−2^, while that of C_3_ plants ranges from 2.80 to 3.64 g·m^−2^ [45]. Ghannoum et al. reported a N_area_ range of 0.48 to 1.23 g·m^−2^ for C_4_ NADP-ME grasses [46]. An analysis by Kattge et al. reported an average of 0.93 ± 1.45 g·m^−2^ for 232 C_4_ grass species [47]. Compared to the above research reports, the present study reported leaf N values within the normal range for *M. condensatus* despite the low soil N content. In addition, it should be noted that the soil N in the BL was about 10% of that in SLG; however, the N_area_ reached 70% of what was recorded in SLG (Table 3). This seems to indicate that *M. condensatus* has a unique nitrogen acquisition conservation strategy in extreme N-limited habitats.

Values of A_max_ for *M. condensatus* (18.81 ± 3.72 µmol·m^−2^·s^−1^) in BL were lower than in SLG and SLUC (Table 1). However, it should be noted that *M. condensatus* achieved 75% A_max_ compared to that in SLG despite low soil N conditions. Generally, C_4_ grasses have a high capacity for photosynthesis. Kattge et al. reported an A_max_ value of 19.78 ± 1.58 µmol·m^−2^·s^−1^ based on 97 species of C_4_ grass in various conditions [47]. However, the Amax of *M. condensatus* (18.81 ± 3.72 µmol·m^−2^·s^−1^) in BL was not drastically lower than this value. All these above indicated that the photosynthetic advantages of *M. condensatus* in Miyake.

To further evaluate the photosynthetic adaptive advantage of *M. condensatus* in the volcanically devastated site, its photosynthetic activity was compared to those of plants growing in other nutrient-poor habitats (e.g., sand dunes, glacier retreated sites, volcanic deserts). A recent study in Australia’s sand dunes compared the photosynthetic activity of several species across three different successional stages, including the early stage of primary succession [22]. Seven pioneer species (not including C_4_ grass species) growing in the early stage of primary succession (plant growth is limited by N) had A_max_ values ranging from 11.3 to 17.8 µmol·m^−2^·s^−1^ [22]. The highest A_max_ value was reported for the native shrub species, *Acacia rostellifera*. Compared to the species monitored by Guilherme Pereira et al. [22], *M. condensatus* appears to have a higher photosynthetic capacity in the early stage of primary succession. However, there are no comparable studies which investigated photosynthesis of C_4_ plants in volcanic deserts. In terms of C_3_ plants, including trees, there are a few studies comparable to our data. Choi et al. reported that the A_max_ value for *A. sieboldiana* (an N-fixing C_3_ tree species) at Miyake-jima Island [12], in a site with low exposure to the disturbances from the 2000 eruption, was 12 µmol·m^−2^·s^−1^. This value is lower than that recorded for *M. condensatus,* even though the study site was highly disturbed and had low soil N. In the volcanically devastated site of Mt. Fuji, Japan, Sakata et al. reported the photosynthetic rate of two C_3_ herb species, *Reynoutria japonica* and *Aconogonum weyrichii* [43]. Both species inhabited an old volcanic desert (last eruption at 1707) exposed to heavy wind, and they had A_max_ values of 18.60 and 22.70 µmol·m^−2^·s^−1^ for *R. japonica* and *A. weyrichii,* respectively. On the other hand, the A_max_ value of *M. condensatus* appears to be higher than that of *Metrosideros polymorpha*, a C_3_ tree species in Hawaii [44]. The A_max_ values of *M. polymorpha* species growing on a 26-year-old lava flow (from an eruption in 1959) and on a 195-year-old lava flow (from an eruption in 1790) in the Kilauea Iki crater were 8.68 µmol·m^−2^·s^−1^ and 8.54 µmol·m^−2^·s^−1^, respectively [3,44]. These comparisons support the finding that *M. condensatus* has a higher photosynthetic capacity in nutrient-poor habitats.

PNUE describes leaf photosynthetic economics, and plants with higher PNUE values are regarded as being more adaptive to N-deplete habitats. In general, C_4_ plants have higher PNUE than C_3_ plants [48,49,50]. Kattge et al. [47] reported the average PNUE value of 80 C_4_ grasses as 263.34 ± 23.23 μmol·CO_2_ mol·N^−1^·s^−1^, which is similar to the value recorded for *M. condensatus* in the present study. We also found that there was no significant difference in PNUE among three study sites. This indicated that the advantages in N use strategy of *M. condensatus.* However, there are no reported studies on the PNUE of species growing on similar nutrient deplete habitats; hence, there is no basis for comparison with the PNUE of *M. condensatus*. This higher PNUE of *M. condensatus* appears to be primarily a result of its C_4_ pathway [46], which can eliminate photorespiration by increasing CO_2_ levels in the vicinity of the enzyme Rubisco. However, to fully understand the relative adaptability to N poor habitat among C_4_ grass, further comparative studies are necessary.

### 3.2. Physiological Cause for the Variation in PNUE

**Figure A1:**
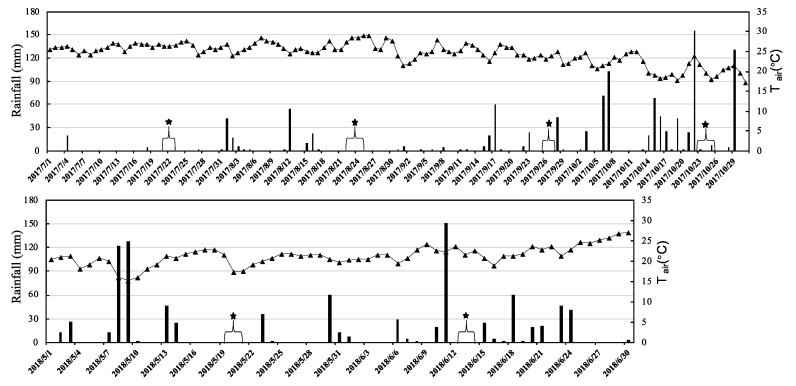
Daily rainfall (bars) and daily average air temperature (T air) (solid line) from June 2017 to July 2018. Pentagonal star represents the survey implementation day.

The GLMM results showed that there were significant negative effects of STN on PNUE, positive effect on N_area_ and A_max_, and no effect on LMA (Table 4). This is consistent with the conclusions of several preceding studies that a higher PNUE facilitates the establishment of plants [46,50,51]. However, in the present study, the effect of the different sites on PNUE values was not significant (Table 2 and Table 3). This is because PNUE is the ratio of A_max_ to N_area_ and the common trend observed in them as a result of soil N levels may be responsible for the non-significant difference in PNUE values between the three study sites. As for the physiological causes of PNUE variation, numerous studies focused on inter-species differences in PNUE suggest that an increase in PNUE with increasing LMA is caused by decreasing photosynthetic rates and lower N partitioning into Rubisco compared to higher N partitioning into cell walls [52,53,54]. However, in this study (about a single species), it was discovered that LMA did not significantly reduce PNUE (Table 3). This is consistent with a previous study, which reported similar PNUE but different LMA values, 40–50 g·m^−2^ and 15–20 g·m^−2^ in the leaves of *Chenopodium album* grown in low nutrient and high average light intensity, and those grown in high nutrient and low average light intensity, respectively. In addition, it was observed that the main factor affecting LMA in the present study was the light condition (Table 4). Hikosaka (2004 for review) [31] also suggested that within a single species, differences in PNUE do not occur when LMA changes with growth light or nutrient availability. Therefore, in evaluating the physiological factors responsible for the variation in PNUE in *M. condensatus*, it is necessary to investigate the relationship between PNUE and the allocation of N among the major foliar N fractions. In addition, GLMM shows the strong positive relationship between precipitation and PNUE. During the growing season, Amax, PNUE showed strong monthly changes, and relative values among the three habitats in each month also varied (Figure 1, Table 2). In BL, Amax increased in June and October (Figure 1d), and the PNUE value in these two months was also markedly higher among the three habitats (Figure 1c). All the changes observed in June and October occurred when measurements were carried out after rainfall. The rainfall in June and October were higher than in other months (Figure A1, Appendix A). These are consistent with the previous finding that temporal rainfall may help plant growth [55,56], and indicate that *M. condensatus* in BL are more sensitive to water deficits. Water deficit is another factor that limits growth, and probably had a large effect on *M. condensatus* grown in newborn soil. In this study, under water stress, *M. condensatus* could not perform all its photosynthetic abilities. Therefore, in the Miyakejima volcanically devastated site, the photosynthetic capacity of *M. condensatus* is achieved when there is a sufficient water supply. The site-specific pattern of *M. condensatus* responses to rainfall suggests that rain may be an important contributor to the growth and survival of *M. condensatus* on the volcanically devastated site.

### 3.3. Physiological Responses to Light Availability

Light availability gradients affect leaf traits, which determine leaf carbon acquisition [57,58], and previous studies have demonstrated that leaves that inhabit low-light environments exhibit shade-acclimatized traits in the long run [59,60]. Results from this study indicated that under low light environments (SLUC), *M. condensatus* had significantly lower LMA and LCP (Table 2), but higher Fv/Fm than those exposed to higher light intensities. The GLMM shows a strong positive relationship between canopy openness and Fv/Fm. These results demonstrate that *M. condensatus* acclimatized to the shade by decreasing LCP and by increasing its ability to capture light (higher Fv/Fm). The higher Fv/Fm observed in SLUC (Table 2, Figure 1) suggests that *M. condensatus* leaves are more efficient at trapping light in the pigment range of PSII under low light conditions [61], enabling them to use light during the constant low-light periods in the SLUC. In addition, Fv/Fm can provide insights on the ability of plants to tolerate environmental stresses and the extent to which those stresses can damage their photosynthetic apparatus [61]. Results from this study indicated that extremely low soil N did not significantly affect Fv/Fm (Table 3). This also proves the high resistance of the species to environmental stress. Furthermore, reduction in LMA is a typical shade acclimatization response, which can improve the light harvested per unit of resource invested in the construction of photosynthetic tissue [59,62].

Although the capacity to photosynthesize and grow under shaded conditions has been proven in this study, due to the limitation of the actual light condition, the actual photosynthesis rate in SLUC was not high (Figure 2). The low A_actual_ in SLUC indicates that the productivity of *M. condensatus* is low under low-light conditions. The lower productivity of *M. condensatus* under the pioneer trees of *A. sieboldiana* can explain the successional change from grassland consisting of *M. condensatus* to shrub land comprising *A. sieboldiana* and other tree species [42].

## 4. Materials and Methods

This study was conducted between 2017 and 2018 on a volcanically devastated site (17 and 18 years after the eruption). At the time of this study, the landscape of the area was composed of bare land, *M. condensatus* grasslands, and *A. sieboldiana* shrubs.

### 4.1. Research Area

This study was conducted at the Miyake-jima active volcanic island, Japan (34°05′ N, 139°55′ E), which covers an area of 55.44 km² and has an altitude of 775.1 m a.s.l. (Mt. Oyama). The island has a humid, temperate climate, with an average annual temperature of 17.7 °C, with the average temperatures of the hottest and the coolest month being 26.2 °C (August) and 9.6 °C (February), respectively. The annual precipitation is 2953.6 mm, and the average precipitation for the months with the minimum and maximum rainfall are 140.3 mm (December) and 383.3 mm (October), respectively [63]. At the time of this study, there were no current effects of volcanic gas as the emissions had already ceased.

### 4.2. Experimental Design and Measurements

**Figure A2:**
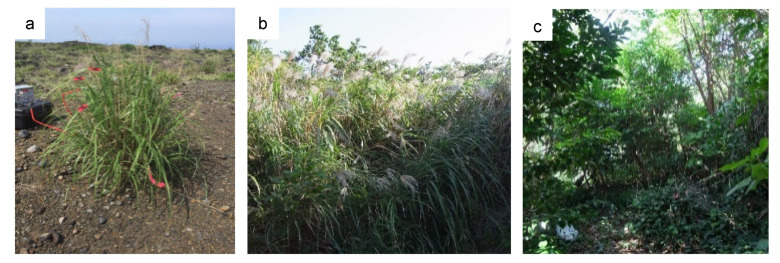
Landscape of bare land (**a**) and shrub land gap (**b**), shrub land under canopy (**c**).

To examine the effects of environment conditions on the leaf traits of *M. condensatus*, three study sites in different successional stages were selected: bare land (BL) with *M. condensatus* patches (Figure A2a), a site with a relatively open tree canopy (SLG: shrub land gap), and a site with a closed canopy (SLUC: shrub land under canopy) (Figure A2b,c). In BL and SLG *M. condensatus* were the absolutely dominant species. The other species such as *A. sieboldiana* (N-fixing species) and *F. japonica* var. *hachidyoensis* (a perennial herb) were also existed sparsely. In SLG, the upper leaves of *M. condensatus* were almost fully exposed to ambient sunlight, while in SLUC, *M. condensatus* was sparsely distributed under the closed canopies, and some dead *M. condensatus* were found. The recovery of vegetation in the study area proceeded in a direction from lower to higher elevation, and from further away from the Oyama crater to closer to the crater [54]. This recovery process may be caused by many factors, such as heavy wind and seed dispersal, the initial damage of volcanic ash and toxic gas [54]. Thus, the three study sites fully reflected the recovery of the vegetation. In addition, differences in actual air temperature and wind caused by changes in altitude may also have affected plant growth in the study site.

**Figure A3:**
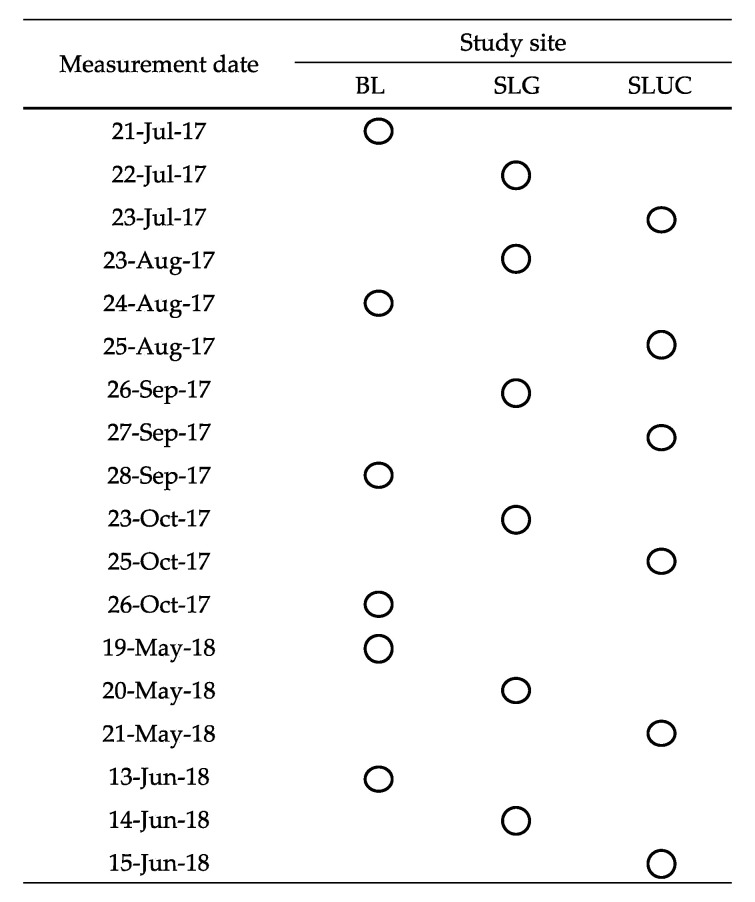
The specific measurement date in bare land (BL), shrub land gap (SLG) and shrub land under canopy (SLUC).

In each study site, eight *M. condensatus* plants were selected for the measurement of leaf traits. The *M. condensatus* selected in BL were between 0.5 and 1.5 m in height. The distance between the selected plants was at least 5 m. In SLG and SLUC, the plants were between 1.8 and 2.3 m in height. In total, 144 leaf samples were measured. In each study site, leaf traits were measured at an interval of 1 month from July to October 2017 and from May to June 2018 (6 months in total) which contained the entire growing season of *M. condensatus*. The specific measurement time for each study site is shown in Figure A3. We measured a total of 48 leaves (eight leaves every month) at each study site. All study sites were measured within 3 days of each other.

### 4.3. Gas Exchange Parameters

To measure the activities of healthy leaves located in the upper part of each sample, the second or third (counted from the top of the shoot) fully expanded leaves were selected every month. Based on continuous field observations (every month from May to October 2018), it was discovered that the leaves of *M. condensatus* in Miyake-jima Islands had a relatively short leaf life span (4 months), and that the production of new leaves was continuous throughout the growing seasons. The relative position of leaves became sequentially lower with the progression of seasons; therefore, leaf samples were collected from the same position in the plant for measurement to make sure they have similar leaf age. In addition, the sampled leaves were evaluated for the determination of LMA and leaf N every month. Intelligent portable photosynthesis system LCPro + (ADC BioScientific, UK) was used to calculate the measurement of gas exchange. At each study site, eightleaves (one leaf per plant) were measured per month. Measurements were taken from the leaves in the middle, and all gas exchange parameters were measured between 9:00 a.m. and 11:00 a.m. During all of the measurements, the chamber CO_2_ concentration, temperature, and vapor pressure deficit (VPD) were maintained at 420 μmol·mol^−1^, 25 °C, and 1.0 kPa, respectively. Light-response curves were determined at irradiances between 2000 and 0 µmol·m^−2^·s^−1^ using a built-in LED light source in seven PPFD steps and were fitted using a non-rectangular hyperbola model [64]. A_max_, and LCP were calculated using light-response curves. WUE was calculated as the ratio of A_max_ to the E at photosynthetic photon flux density (PPFD) saturation [65].

### 4.4. Structural and Biochemical Characteristics of Leaves

When the measurements of photosynthetic parameters were completed, the sampled leaves were collected and wiped; the measured areas were cut out and scanned, and the images were processed using ImageJ software (National Institutes of Health, Bethesda, MD, USA, imagej.nih.gov/ij) for leaf area. For leaf dry mass/leaf area determination, cut-out leaf samples were dried at 80 °C for 48 h to constant weight and measured for their final weight. The remaining leaf samples were also dried for the determination of leaf N using an NC analyzer (SUMIGRAPH NC-220F). The PNUE was calculated as A_max_ divided by N_area_ (PNUE, μmol·CO_2_ mol·N^−1^·s^−1^ = A_max_ (µmol·m^−2^·s^−1^)/(1/14N_area_) [52].

### 4.5. Chlorophyll Fluorescence

While measuring gas exchange parameters, three light-adapted leaves (the middle part) of each plant were selected for the measurement of chlorophyll fluorescence using MINI-PAM-II-Walz. The minimum fluorescence (F0) and maximum fluorescence (Fm) were determined following a 30 min dark adaptation using leaf clips, in order to calculate the maximum photochemical efficiency of PSII (Fv/Fm).

### 4.6. Rainfall Distribution before Measurements

To assess the effects of precipitation on the leaf traits of *M. condensatus*, the rainfall distribution before the measurement day of each month was analyzed. We used the 3-day integrated rainfall from before the photosynthetic measurements, from the daily rainfall data from the Japan Meteorological Agency (2019) [63]. The highest levels of rainfall (before the measurement day) were recorded in June and October (Figure A1).

### 4.7. Soil Measurements

In October 2017, soil samples from the root zones of the sampled *M. condensatus* plants were collected using 100 mL core samplers of 5 cm depth for the determination of the soil properties of the study sites. Surface soils (5 cm in depth) were collected because *M. condensatus* develops its root system on the ground surface and its fine roots are concentrated in shallow soils. Three cores were sampled from the soil around the root crown (approximately 10 cm away from the root crown) and were subsequently combined and mixed. After air-drying, the samples were passed through 2 mm and 0.5 mm sieves, respectively, and the roots were removed. Soil samples between 0.5 mm and 2 mm were ground to sizes lesser than 0.5 mm. The soil total C (STC) and STN were analyzed with SUMIGRAPH NC-220F, using samples smaller than 0.5 mm.

### 4.8. Light Measurements

Every time we measure photosynthetic parameters, Hemispherical photographs were taken using a Nikon Coolpix 990 and a Nikon FC-E8 Fisheye Converter (Nikon, Tokyo, Japan). Photographs were taken above each plant (totally, 48 were taken in each study site) and were analyzed to calculate canopy openness using Gap Light Analyzer (GLA Version 2.0) [66]. Canopy openness was defined as the fraction of open sky in the hemisphere that was visible from a point beneath the canopy and was used as an index of the light available to the *M. condensatus* plants.

A representative location was selected in the three sites for the measurement of photosynthetic photon flux density (PPFD) at sunny or cloudy days, with the aid of IKS-27 sunshine sensors (KOITO MANUFACTURING CO., LTD, Yokohama, Japan) which were mounted on poles at a height of 1 m. The measurements were done simultaneously in the three sites between 8:00 a.m. and 4:00 p.m. in the month of August 2016 and reading of data was done every 30 s. We used maximum daily PPFD for each site and light-response curve for each leaf sample (48 samples in each study site) to estimate the actual photosynthetic rates (A_actual_). This parameter can be used to explain the photosynthetic rate in natural light condition.

### 4.9. Data Analysis

All analyses were carried out using the statistical package R version 3.6.0 [67]. For each leaf traits variable, data were analyzed by two-way ANOVA, with study sites and month as main fixed factors plus a site × month interaction term (Table 2). The site differences of annual mean value of each leaf traits were compared (Table 3). Prior to analyses, the assumptions of normality and homogeneity of variances were tested using Shapiro–Wilk and Bartlett tests. For leaf traits that meets homogeneity of variance and normal distribution (E, N_area_, LMA, A_max_, PNUE), one-way ANOVA was applied to assess the differences of them followed by Tukey’s post hoc test used for multiple comparisons between the sites. A non-parametric Kruskal–Wallis test was performed to check for differences in environmental factors and some leaf traits (Fv/Fm, LCP, and WUE) among the three study sites. Steel Dwass post hoc test was performed to detect significant differences in the mean value of environmental factors, Fv/Fm, LCP, and WUE. The generalized linear mixed model (GLMM) with the package lme4 [68] was used to assess the effect of environment factors on leaf traits. The predictors (fixed effects) including the soil N, canopy openness, temperature and precipitation (3-day integrated rainfall), while the response variables were leaf traits (Fv/Fm, E, N_area_, LCP LMA, and A_max_, WUE, PNUE), respectively. The study site and month were included as random effect factors. Marginal and conditional R² were calculated.

## 5. Conclusions

We investigated the leaf traits of *M. condensatus*, a non-N-fixing pioneer species that grows in Miyake-jima, a volcanically devastated site. The results showed that compared to previous studies and internal comparison of Miyake Island, *M. condensatus* could maintain a relatively higher N_area_, A_max_ despite under extreme N-deficient conditions. This is partly as a result of its higher PNUE. PNUE in *M. condensatus* appears to be high even for a C_4_ grass; this is a characteristic necessary for its successful growth in N-deficient soils. The higher PNUE of *M. condensatus* may be an intrinsic function of its C_4_ pathway. We also found that PNUE values in BL were higher than in the other two habitats in June and October. Considering the level of rainfall before our measurements, the higher PNUE in BL was related to rainfall.

## Figures and Tables

**Table 1:** Location and morphometric characteristics of bare land (BL), shrub land gap (SLG) and shrub land under canopy (SLUC). Different letters indicate significant differences between the study sites revealed by Steel Dwass post hoc test at a significance level *p* < 0.05 after Kruskal–Wallis test. Abbreviations are meters above sea level (m a.s.l); photosynthetic photon flux density (PPFD); soil total N (STN) (n = 30); soil total carbon (STC) (n = 30); Canopy openness (n = 48).

Factor	BL	GL	SL
Location	34°04.689′ N, 139°30.816′ E	34°04.790′ N, 139°30.245′ E	34°04.790′ N, 139°30.245′ E
Altitude (m a.s.l)	500	400	400
Ash depth (cm)	30–35	30–35	30–35
Air temperature (°C)	25.54	26.16	25.64
Maximum PPFD (µmol·m^−2^·s^−1^)	1227	531	102
Canopy openness (%)	74 ± 0.5 a	68 ± 1.3 a	24 ± 1.4 b
STN (%)	0.02 ± 0.002 b	0.24 ± 0.02 a	0.30 ± 0.01 a
STC (%)	0.20 ± 0.03 b	4.54 ± 0.68 a	5.78 ± 0.42 a

**Table 2:** The F values of two way-ANOVA for effects of site, month and their interactions on each leaf traits (n = 144) of *M. condensatus*. Abbreviations are maximum quantum yield of PSII (Fv/Fm); transpiration rate (E); leaf N content (Narea); light compensation point (LCP); leaf mass per area (LMA); light-saturated photosynthetic rate (Amax); water use efficiency (WUE); photosynthetic N use efficiency (PNUE). Significance of the coefficients: * *p* < 0.05, ** *p* < 0.01, *** *p* < 0.001.

Leaf Traits	Site	Month	Site
Fv/Fm	133.359 ***	18.826 ***	5.682 ***
E (mmol^−1^·H_2_O m^−2^·s^−1^)	44.21 ***	31.2 ***	14.32 ***
N_area_ (g·m^−2^)	40.372 ***	2.31 *	2.796 **
LCP (µmol·m^−2^·s^−1^)	39.24 ***	8.59 ***	2.95 **
LMA (g·m^−2^)	140.834 ***	8.301 ***	2.901 **
A_max_ (µmol·m^−2^·s^−1^)	32.03 ***	1.666	2.837 **
WUE (μmol·mmol^−1^)	2.76	3.82 **	2.779 **
PNUE (μmol·CO_2_ mol·N^−1^·s^−1^)	1.624	0.927	3.067 **

**Table 3:** Leaf traits of *M. condensatus* (Mean value ± SE; n = 48) in bare land (BL), shrub land gap (SLG) and shrub land under canopy (SLUC). Abbreviations are maximum quantum yield of PSII (Fv/Fm); transpiration rate (E); leaf N content (N_area_); light compensation point (LCP); leaf mass per area (LMA); light-saturated photosynthetic rate (A_max_); water use efficiency (WUE); photosynthetic N use efficiency (PNUE). For E, N_area_, LMA, A_max_, and PNUE, different letters indicate significant differences between the study sites revealed by Tukey’s post hoc test at a significance level *p* < 0.05 after one-way ANOVA. For Fv/Fm, LCP and WUE, different letters indicate significant differences between the study sites revealed by Steel Dwass post hoc test at a significance level *p* < 0.05 after Kruskal–Wallis test.

Leaf Traits	BL	SLG	SLUC
Fv/Fm	0.69 ± 0.05 c	0.75 ± 0.03 b	0.77 ± 0.05 a
E (mmol^−1^·H_2_O·m^−2^·s^−1^)	1.51 ± 0.05 b	2.22 ± 0.06 a	2.05 ± 0.07 a
N_area_ (g·m^−2^)	1.07 ± 0.25 c	1.47 ± 0.22 a	1.24 ± 0.22 b
LCP (µmol·m^−2^·s^−1^)	27.08 ± 3.53 a	32.54 ± 1.89 a	16.74 ± 1.02 b
LMA (g·m^−2^)	89.29 ± 11.11 a	79.04 ± 8.61 b	60.03 ± 10.98 c
A_max_ (µmol·m^−2^·s^−1^)	18.81 ± 3.72 c	25.44 ± 4.91 a	23.56 ± 4.65 b
WUE (μmol·mmol^−1^)	12.29 ± 1.17 a	9.39 ± 0.21 b	9.73 ± 0.20 b
PNUE (μmol·CO_2_ mol·N^−1^·s^−1^)	258.79 ± 82.75 a	247.49 ± 65.66 a	272.56 ± 69.82 a

**Table 4:** Effect of environment factors on each leaf traits (n = 144) of *M. condensatus* (generalized linear mixed model—GLMM). Response variables are maximum quantum yield of PSII (Fv/Fm); transpiration rate (E); leaf N content (Narea); light compensation point (LCP); leaf mass per area (LMA); light-saturated photosynthetic rate (Amax); water use efficiency (WUE); photosynthetic N use efficiency (PNUE), respectively. MR² Significance of the coefficients: ns *p* > 0.05, * *p* < 0.05, ** *p* < 0.01, *** *p* < 0.001.

Traits		Estimate	Std. Error	*p*	MR²	CR²
Fv/Fm	Canopy openness	−0.0009	0.0004	*	0.22	0.63
	STN	0.0091	0.0398	ns		
	Precipitation	−0.00003	0.0001	ns		
	Temperature	0.0015	0.0013	ns		
E (mmol^−1^·H_2_O·m^−2^·s^−1^)	Canopy openness	−0.0106	0.0057	ns	0.18	0.70
	STN	−0.3054	0.6187	ns		
	Precipitation	0.0074	0.0013	***		
	Temperature	0.0123	0.0218	ns		
N_area_ (g·m^−2^)	Canopy openness	0.0028	0.0019	ns	0.35	0.63
	STN	1.5840	0.2406	***		
	Precipitation	−0.0004	0.0004	ns		
	Temperature	−0.0103	0.0081	ns		
LCP (µmol·m^−2^·s^−1^)	Canopy openness	0.8896	0.1060	***	0.50	0.95
	STN	−3.4123	10.0378	ns		
	Precipitation	−0.0098	0.0183	ns		
	Temperature	0.5708	0.2922	ns		
LMA (g·m^−2^)	Canopy openness	0.6461	0.0793	***	0.75	0.85
	STN	−15.6300	10.1579	ns		
	Precipitation	−0.0223	0.0192	ns		
	Temperature	0.6693	0.3565	ns		
A_max_ (µmol·m^−2^·s^−1^)	Canopy openness	0.0181	0.0376	ns	0.539	0.802
	STN	40.6600	4.7130	***		
	Precipitation	0.0247	0.0087	**		
	Temperature	0.2123	0.1616	ns		
WUE (μmol·mmol^−1^)	Canopy openness	0.0064	0.0098	ns	0.04	0.42
	STN	0.2923	1.7096	ns		
	Precipitation	−0.0080	0.0050	ns		
	Temperature	0.1058	0.0810	ns		
PNUE (μmol·CO_2_ mol·N^−1^·s^−1^)	Canopy openness	−1.9851	0.4008	**	0.34	0.45
	STN	−404.9090	60.8571	***		
	Precipitation	0.4855	0.1341	***		
	Temperature	3.0690	2.3935	ns
